# Nucleic Acid, Antibody, and Virus Culture Methods to Detect Xenotropic MLV-Related Virus in Human Blood Samples

**DOI:** 10.1155/2011/272193

**Published:** 2011-11-17

**Authors:** M. F. Kearney, K. Lee, R. K. Bagni, A. Wiegand, J. Spindler, F. Maldarelli, P. A. Pinto, W. M. Linehan, C. D. Vocke, K. A. Delviks-Frankenberry, R. W. deVere White, G. Q. Del Prete, J. W. Mellors, J. D. Lifson, V. N. KewalRamani, V. K. Pathak, J. M. Coffin, S. F. J. Le Grice

**Affiliations:** ^1^HIV Drug Resistance Program, National Cancer Institute at Frederick, Frederick, MD 21702-1201, USA; ^2^Protein Expression Laboratory, SAIC-Frederick, Inc., NCI-Frederick, Frederick, MD 21702, USA; ^3^Urologic Oncology Branch, National Cancer Institute, Bethesda, MD 20892, USA; ^4^UC Davis Cancer Center, Sacramento, CA 95817, USA; ^5^AIDS and Cancer Virus Program, SAIC-Frederick, Inc., National Cancer Institute, Frederick, MD 21702, USA; ^6^Department of Medicine, University of Pittsburgh, Pittsburgh, PA 15260, USA; ^7^Department of Molecular Biology and Microbiology, Tufts University, Boston, MA 02155, USA

## Abstract

The MLV-related retrovirus, XMRV, was recently identified and reported to be associated with both prostate cancer and chronic fatigue syndrome. At the National Cancer Institute-Frederick, MD (NCI-Frederick), we developed highly sensitive methods to detect XMRV nucleic acids, antibodies, and replication competent virus. Analysis of XMRV-spiked samples and/or specimens from two pigtail macaques experimentally inoculated with 22Rv1 cell-derived XMRV confirmed the ability of the assays used to detect XMRV RNA and DNA, and culture isolatable virus when present, along with XMRV reactive antibody responses. Using these assays, we did not detect evidence of XMRV in blood samples (*N* = 134) or prostate specimens (*N* = 19) from two independent cohorts of patients with prostate cancer. Previous studies detected XMRV in prostate tissues. In the present study, we primarily investigated the levels of XMRV in blood plasma samples collected from patients with prostate cancer. These results demonstrate that while XMRV-related assays developed at the NCI-Frederick can readily measure XMRV nucleic acids, antibodies, and replication competent virus, no evidence of XMRV was found in the blood of patients with prostate cancer.

## 1. Introduction

Xenotropic murine leukemia virus-related virus (XMRV) is a recently discovered gammaretrovirus reportedly associated with prostate cancer and chronic fatigue syndrome (CFS) [1, 2]. The discovery of XMRV arose from studies investigating a potential viral cause for diseases in patients with an *RNAseL* gene variant. This genotype, which is observed in a varying subset of patients in cohorts with prostate cancer [1, 3–8], has been associated with impairment of innate immune responses to viral infections [5]. Seeking an etiologically significant viral infection associated with impaired *RNAse L*-dependent responses, Urisman et al. first identified XMRV in 2006 in a cohort of prostate cancer patients [2]. The association of XMRV with prostate cancer, but not its association with the *RNAseL* variant, was corroborated by Schlaberg et al. in 2009 [9]. The prostate cancer studies were followed by a report from Lombardi et al. presenting evidence for XMRV infection in 67% of individuals with severe CFS, compared to 3.7% of healthy individuals [1]. These high reported frequencies of XMRV infection and putative linkage to a debilitating illness prompted concerns about the possibility of a new, widespread retroviral epidemic and stimulated additional research towards determining the prevalence of XMRV infection in different populations worldwide.

Several studies supporting high prevalence of XMRV infection followed. For example, Arnold et al. detected anti-XMRV antibodies in 27% of individuals with prostate cancer [10], Schlaberg et al. found XMRV nucleic acid in 23% of prostate cancers and 4% of controls [11], and Danielson et al. detected XMRV in 22.8% of extracted prostate tissues from individuals who had radical prostatectomies [12]. However, controversy arose when other laboratories could not demonstrate comparable findings in similar cohorts not only in the US [13] but in Germany [14], The Netherlands [15], and England [16, 17]. Adding to the controversy, Lo et al. reported the presence of mouse retroviral sequences, but not XMRV, in 86.5% of CFS patients [18]. Claims were made that such findings supported the association of XMRV infection with CFS, complicating an already controversial field. 

Several factors were speculatively proposed to contribute to the differential detection of XMRV/MLVs by different laboratories. It was suggested that inconsistencies in detection of XMRV/MLVs in patient samples could result from varied prevalence of infection in different populations, differing criteria for patient selection, and differing detection methodologies utilized [19]. It was also proposed that virus levels may be chronically low or episodic in patient plasma or tissues, making virus detection difficult [19]. Adding to the complexity, detection of XMRV by PCR is highly susceptible to false positive results due to the very close genetic relationship of XMRV with endogenous MLVs and the high prevalence of contaminating mouse genomic DNA in many specimens [20, 21]. Indeed, studies have suggested that XMRV detection is the result of laboratory contamination from infected cell lines [22–25] or contaminated reagents [26]. Further suggestions of laboratory contamination came after publication of a study by Paprotka et al. [25], showing that XMRV originated in a human cancer cell line generated by passaging prostate cancer cells through immunocompromised mice. This result indicates that XMRV could not have entered the human population until recently, yet was already being reported as prevalent in a sizeable fraction of prostatic cancers. Furthermore, it showed that most “XMRV-specific” detection assays could, in fact, detect one or the other of the two parental proviruses (PreXMRV-1 and 2) that gave rise to XMRV and are endogenous to some inbred and wild mice. In assessing this situation, it became clear that to rule out false positive results and reliably detect XMRV infection, one must apply several diagnostic methods used in conjunction with known positive and negative controls. 

At the NCI-Frederick, we sought to help clarify the XMRV controversy by generating multiple assays, including rigorous methods to measure antibodies to XMRV through ELISA-based methods, to quantify XMRV proviral DNA and viral RNA through quantitative PCR and RT-PCR methods, and to measure infectious virus by viral isolation cultures using an indicator cell line system. We characterized these assays using available positive and negative control samples, including spiked samples and specimens from two pigtail macaques experimentally inoculated with XMRV. We then applied these methods to specimens from two cohorts of prostate cancer patients to determine the levels of XMRV in their blood. Overall, we observed a high level of concordance between detection methods and were able to rule out false positive results by applying multiple assays on the same patient samples. Applying this approach, we did not find evidence of XMRV infection in any of the prostate cancer patient-derived specimens studied.

## 2. Methods

### 2.1. Clinical Prostate Cancer Samples

The XMRV detection assays developed at the NCI-Frederick were applied to samples collected from two cohorts of prostate cancer patients. In total, 134 patients were studied. Plasma samples from 108 patients were obtained at the UC Davis Cancer Center. Samples were collected between 2006 and 2010 from prostate cancer patients who were either newly diagnosed, on active treatment, or undergoing post-treatment monitoring. Plasma from all 108 patients was tested for XMRV RNA and antibodies to CA and TM. Institutional Review Board (IRB) approval was obtained from the UC Davis Cancer Center Biorepository, and all study subjects provided written informed consent.

Samples from an additional 26 recently diagnosed prostate cancer patients were obtained from the Urologic Oncology Branch, NIH Clinical Center, Bethesda, MD. All 26 blood samples were tested for the presence of XMRV RNA in plasma and DNA in whole blood. Tests for XMRV proviral DNA were also performed on prostate tissue from 19 of the 26 individuals in this cohort who had radical prostatectomies. Twenty-two of 26 blood samples were tested for antibodies to CA and TM. A subset of 12 samples was tested by virus rescue culture including those that had positive or indeterminate results by X-SCA or ELISA and matched negative controls. The study was approved by the IRB of NCI, NIH, Bethesda, MD, and all study subjects provided written informed consent.

### 2.2. XMRV Nucleic Assay Detection with XMRV Single-Copy Assays (X-SCA)

**Figure 1:**
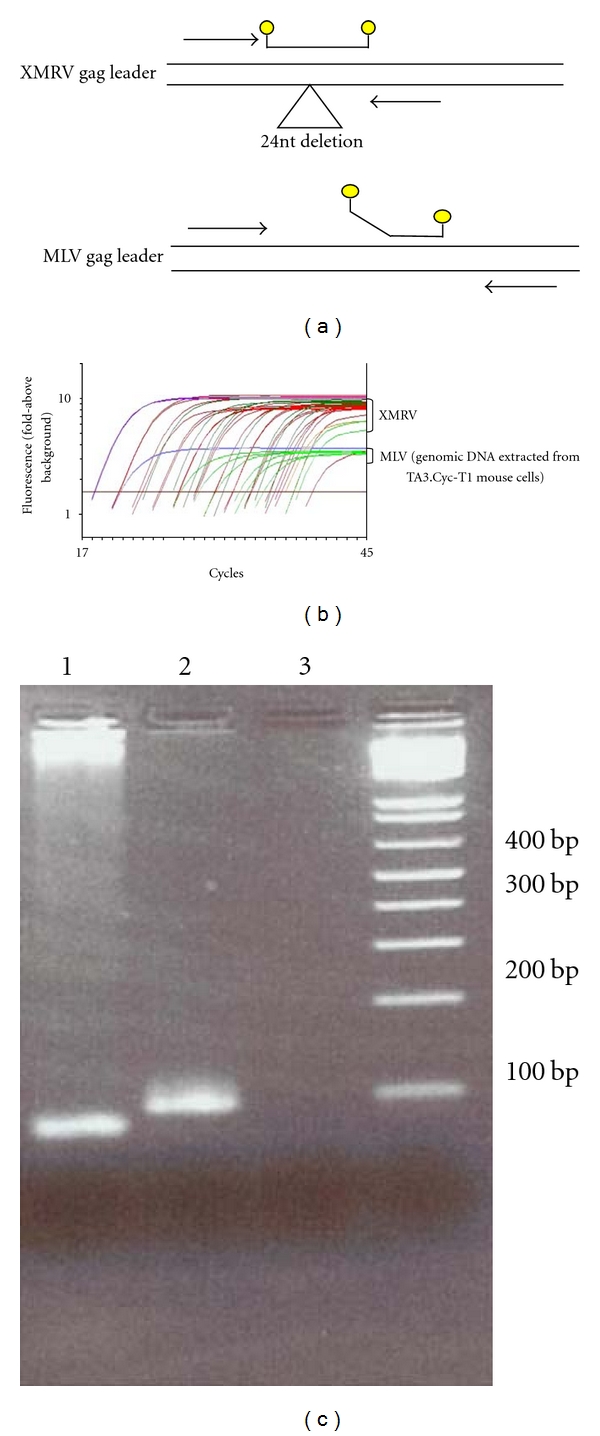
*XMRV single-copy assay (X-SCA)*. X-SCA primers anneal to conserved regions in XMRV/MLV gag leader region while the probe spans a 24 nt deletion in XMRV compared to MLV (a) allowing for differential amplification profiles for XMRV and MLV (b). X-SCA amplification products run on a 2% agarose gel distinguish between the products being amplified since the XMRV product is 24 nt smaller than the MLV product. Lane 1 is the X-SCA product from the XMRV standard curve, Lane 2 is the MLV product from the genomic DNA extracted from TA3.Cyc-T1 mouse cells, and Lane 3 is the “no template” negative control (c).

Similar to the single-copy assay (SCA) for human immunodeficiency virus (HIV) [27], quantitative real-time PCR and RT-PCR assays for detection of XMRV, called XMRV single-copy assays (X-SCA), were developed to quantify XMRV nucleic acid in plasma, whole blood, and cell suspensions obtained from blood or tissue samples. The assays were designed using amplification primers targeting a *gag* leader region conserved between XMRV (as well as PreXMRV-2 [25]) and non-XMRV endogenous MLVs (forward 5-TGTATCAGTTAACCTACCCGAGT-3′, reverse 5-AGACGGGGGCGGGAAGTGTCTC-3′). Consequently, efficient amplification is achieved from both target templates allowing detection of either XMRV or MLVs present in patient samples. The Taqman probe (5′fam-TGG  AGT  GGCTTT  GTT  GGG  GGA  CGA-  tamra3′) used for detection of amplified products was designed to span a signature 24 nucleotide deletion in the XMRV (PreXMRV-2) *gag* leader that differentiates these from all other MLV sequences (Figure 1(a)). In the event that a positive sample is identified by X-SCA, single-genome sequencing should be performed to confirm that the source of amplification was XMRV and not contaminating mouse DNA with a similar gag deletion, such as PreXMRV-2. 

 XMRV RNA was extracted from plasma samples following ultracentrifugation exactly as described for HIV SCA [27] and genomic DNA was extracted and whole blood samples using the Promega genomic DNA Extraction Kit (Cat no. A1120) according to the manufacturer's suggested protocol. Reaction conditions for synthesizing cDNA and measuring RNA copy number were exactly as described previously for HIV SCA [27]. XMRV proviral copy number was determined using the Lightcycler 480 Probes Master (Cat no. 04707494001) according to protocol and by performing 45 cycles of 95°C for 15 seconds, 60°C for 1 minute after an initial 10 minute, 95°C polymerase activation step. Accurate detection of XMRV by X-SCA was verified by testing spiked human blood products [28] and by testing blood samples collected from XMRV inoculated macaques (Del Prete et al., in preparation). Pigtail macaques were experimentally inoculated with XMRV (~4.8 × 10^9^ RNA copy equivalents) prepared from the supernatant of 22Rv1 cells (Lot SP1592, Biological Products Core, AIDS and Cancer Virus Program, SAIC-Frederick, Inc, NCI-Frederick). Plasma and PBMC samples were collected prior to inoculation and through 119 days after inoculation. These pre- and post-inoculation specimens were used as reference control samples in evaluating X-SCA methods for detection of XMRV. Details of the macaque infection study will be reported elsewhere (Del Prete et al. in preparation). Animals were housed and cared for in accordance with American Association for Accreditation of Laboratory Animal Care (AALAC) standards in an AAALAC accredited facility, and all animal procedures were performed according to a protocol approved by the Institutional Animal Care and Use Committee of the National Cancer Institute. Detection of MLV was qualified by extracting mouse genomic DNA from TA3.Cyc-T1 cells using the Promega genomic DNA Extraction Kit (Cat no. A1120) and performing X-SCA in duplicate on dilutions of 3000 to 0.03 cell equivalents.

All patient samples were tested by X-SCA in duplicate or triplicate with equal numbers of no template controls (NTC) to monitor the level of false positives due to either viral or mouse genomic DNA contamination. The level of detection for XMRV nucleic acid in clinical samples was determined by the volume of sample available for testing (100 *μ*L to 3 mL). Therefore, X-SCA sensitivity varied from 0.6 to 20.6 copies/mL of plasma and 0.9–10 copies/mL in whole blood. Because of the high frequency of false positives due to contaminating mouse DNA, we set strict criteria for declaring a sample positive for XMRV, requiring detection of viral sequence in all replicate PCR reactions from the samples being tested. These criteria result in a minimum detection of 1.8–41.2 copies XMRV RNA/mL in plasma and 2.7–30 copies XMRV DNA/mL in whole blood for a positive X-SCA test, depending on the volume of sample being tested. If discordant results are obtained from duplicate or triplicate wells, then the result is considered indeterminate and is repeated where sufficient sample is available.

### 2.3. XMRV Serology

XMRV antigens were prepared in the Protein Expression Laboratory, SAIC-Frederick, MD, as previously described [29]. Purified XMRV antigens were used to develop and optimize ELISA-based protocols (Bagni et al., in preparation). Briefly, purified CA and TM were spotted onto Meso Scale Discovery (MSD) (Gaithersburg, MD) standard 96-well plates at 8 *μ*g/mL and 2 *μ*g/mL, respectively. Samples were diluted 1 : 100 and incubated with individual XMRV antigens. Human antibodies were detected using biotin labeled anti-human IgG (Jackson ImmunoResearch, West Grove, Pa) and MSD-proprietary Sulfo-tagged streptavidin detection reagent and read on a SECTOR Imager 6000 (MSD) plate reader. The XMRV serology assays were qualified with samples obtained from XMRV-inoculated macaques (Del Prete et al., in preparation). Patient samples were considered reactive if the MSD electrochemiluminescent signal (ECL) was at least 50% relative to the ECL signal of the macaque positive control sera. Less reactive patient samples that were at least 2 standard deviations above the average negative human sample were considered indeterminate.

### 2.4. XMRV Culture Detection

The presence of replication-competent XMRV was determined in a virus rescue coculture assay using indicator cells designated DERSE (Detectors of Exogenous Retroviral Sequence Elements) and using expression of a GFP reporter as the readout. DERSE.LiGP cells are a subclone of LNCaP cells (gift from Dr. Francis Ruscetti, NCI) stably transfected with pBabe.iGFP-puro and screened for susceptibility to XMRV infection (Lee et al., in preparation). pBabe.iGFP-puro is an MLV proviral vector that encodes an intron-interrupted reporter GFP gene and is only expressed after mobilization by an infecting gammaretrovirus for a second round of infection of DERSE.LiGP cells. Similar MLV vectors that only express a reporter after being propagated in infection have been described previously using HEK293 cells [30]. The DERSE.LiGP assay will detect any MLV-related viruses that are capable of replicating in human prostate cancer cells. Virus replication can be detected by monitoring GFP-positive cells either by fluorescence microscopy or FACS analysis. 

DERSE.LiGP indicator cells were maintained in Roswell Park Memorial Institute (RPMI) media 1640 (Invitrogen) supplemented with 15% fetal bovine serum (FBS) (Hyclone), 1x Pen/Strep/Glutamine (100 U/mL Penicillin, 0.1 mg/mL Streptomycin, and 0.292 mg/mL Glutamine, Invitrogen) and 1 *μ*g/mL puromycin (Calbiochem). DERSE.LiGP cells were plated at 1 × 10^5^ cells/well in a 24-well tissue culture plate one day before infection. As a positive control, 22Rv1 cell supernatants were diluted in RPMI media and added to cells the next day in the presence of 5 *μ*g/mL of polybrene [31]. Culture medium was refreshed the following day by replacement or splitting cells at a 1 : 3 ratio depending on cell density. Although GFP can be detected in positive control samples within 3 days of infection, to maximize sensitivity for detection of low levels of virus, DERSE.LiGP cells exposed to clinical specimens were maintained in culture for at least two weeks and observed at intervals by fluorescence microscopy. After two weeks, cells were resuspended in a 2% paraformaldehyde (PFA) solution and GFP expression was measured by FACS (FACSCalibur, Becton Dickinson), indicative of a spreading infection. While DERSE.LiGP cells are relatively insensitive to heparin, plasma samples containing EDTA are toxic to the cultures. To mitigate toxicity, 200 *μ*L of EDTA containing plasma samples were distributed into Eppendorf tubes in the presence of 7.5 mM CaCl_2_ to neutralize the EDTA and 30 U/mL heparin salt to minimize sample clotting. Tubes were incubated for 4 hrs at 4°C to separate the plasma from residual clotting. Accurate detection of XMRV by virus culture was verified using a dilution series of supernatants from 22Rv1 cells and XMRV-spiked human plasma samples containing approximately 10^7^ to 10 copies of XMRV RNA. Using XMRV-spiked samples, we noted a loss of detection sensitivity of three- to fivefold in EDTA containing plasma samples treated in the above manner. A recent report of XMRV inactivation by human complement may explain in part the loss of infectivity after addition of plasma [24]. Prostate cancer samples with indeterminate results by X-SCA or ELISA were matched with negative samples and tested blinded in the virus culture assay. 

We required that samples test positive for XMRV nucleic acid (RNA or DNA) and by at least one other detect method (immunoassay or culture assay) to be declared positive for XMRV infection.

All reagents developed at the NCI-Frederick and described here are being made available to the extramural research community through the NIH AIDS Research and Reference Reagent Program or AIDS and Cancer Virus Program, SAIC-Frederick, Inc., National Cancer Institute, Frederick.

## 3. Results

### 3.1. Differentiating between XMRV and MLV with X-SCA Probe

The X-SCA probe used for detection of amplified products spans a signature 24 nucleotide deletion in the XMRV [1] and in the PreXMRV-2 [32] *gag* leader that differentiates these from all other MLV sequences (Figure 1(a)). Amplifications of XMRV from 22Rv1 DNA and MLV from mouse genomic DNA (extracted from TA3.CycT1 cells) show that the probe design results in a lower level of plateau fluorescence from non-XMRV MLV templates than from XMRV templates (Figure 1(b)), likely due to inefficient binding and/or degradation of the probe during MLV extension compared to XMRV extension. The result of the probe design is differential amplification profiles for XMRV and MLV, indicating which product is being detected in the assay and the proportions of each if both templates are detected. To confirm the result, the products were run on an agarose gel (Figure 1(c)). The XMRV X-SCA product is 86 nt long and the MLV product 110 nt, easily distinguishable on a 2% agarose gel.

### 3.2. Qualifying XMRV Assay Detection Capabilities with Spiked Human Samples

Assays for detection of XMRV nucleic acid and replication-competent virus were established using XMRV-spiked samples as positive control specimens. To determine the accuracy and sensitivity of X-SCA methods to detect XMRV in human blood products, we tested a full panel of plasma and whole blood samples that were spiked or not spiked with XMRV derived from 22Rv1 cells. The panel was blinded as to which samples were XMRV positive and which were XMRV negative and were provided to us by the XMRV Scientific Research Working Group for testing by X-SCA [28]. Results from the blinded panel of spiked samples were described previously by Simmons et al. [28] and demonstrated that we detected XMRV RNA and proviral DNA using X-SCA with 100% accuracy. The level of sensitivity for detecting XMRV RNA in the spiked plasma panel was limited by the volume of sample tested for XMRV (270 *μ*L) to 3.3 RNA copies/mL. The level of sensitivity for detecting XMRV proviral DNA was a single XMRV-infected 22Rv1 cell in whole blood samples. All unspiked samples were properly reported as negative for XMRV detection indicating a very low rate of false positivity. 

**Figure 2:**
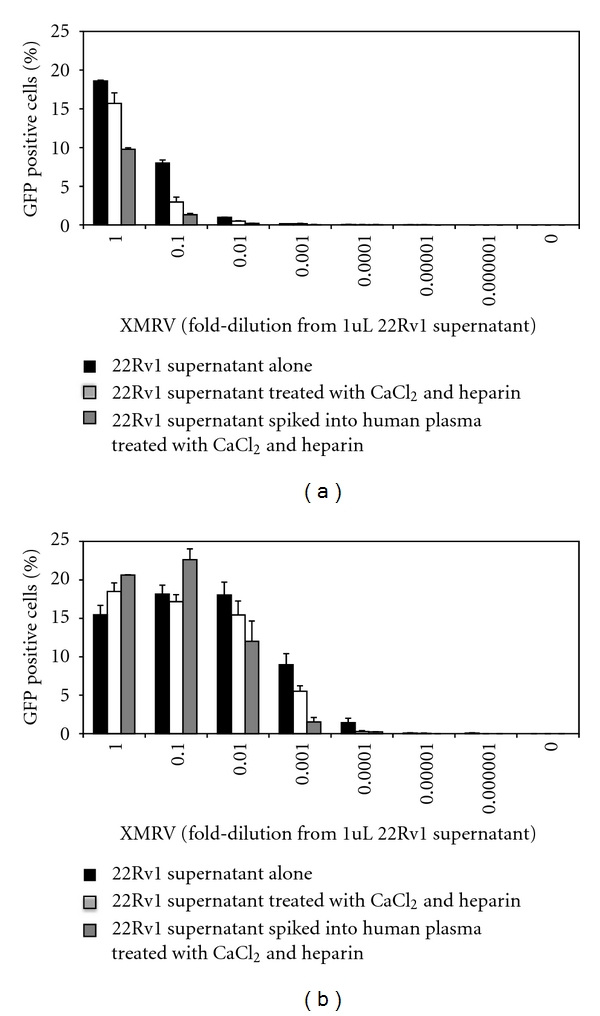
*Verifying XMRV rescue by culturing on DERSE cells with 22Rv1 supernatants and with XMRV-spiked human plasma.* XMRV culturing under the following conditions: (i) 22Rv1 supernatant alone (black bars), (ii) 22Rv1 supernatant treated with CaCl_2_ + heparin (white bars), (iii) 22Rv1 supernatant spiked into human plasma treated with CaCl_2_ + heparin (gray bars). GFP-positive cells were analyzed by FACS at day 4 (a) and day 8 (b).

 The use of DERSE.L-iG-P cells to detect XMRV was verified using 22Rv1 culture supernatants and XMRV-spiked human plasma.  Figure 2 shows the results from virus rescue experiments performed under the following conditions (i) 22Rv1 supernatant alone, (ii) 22Rv1 supernatant treated with CaCl_2_ and heparin, (iii) 22Rv1 supernatant spiked into human plasma treated with CaCl_2_ and heparin. DERSE.LiGP cells treated with EDTA-containing human plasma alone are not viable. Proportions of GFP-positive cells detected by FACS at day 4 and day 8 after infection are shown in Figures 2(a) and 2(b). DERSE.LiGP cells exposed to 0.01 *μ*L of 22Rv1 supernatant were GFP-positive by microscopy within 4 days of infection (Figure 2) demonstrating the sensitivity of this assay for detection of replication competent XMRV. The sensitivity of this detection decreased 3–5-fold in the presence of EDTA-containing plasma samples treated as described above. This decrease could in part be due to the presence of human complement as has been recently reported [24]. Additional days of culture increased the number of GFP-positive cells exposed to virus in the presence or absence of plasma. For this reason, cultures infected with human specimens were carried out for a minimum of two weeks.

### 3.3. Verifying Assay Detection Capabilities with Blood Samples from XMRV-Inoculated Macaques

To validate the specificity of X-SCA and ELISA, we used specimens from two pigtail macaques experimentally inoculated with XMRV. Detailed results from the macaque study will be reported elsewhere (Del Prete et al., in preparation). In short, samples tested by X-SCA revealed that peak viremia was achieved at 5 days after inoculation in one animal and at 13 days in the second (Table 1). By day 28, levels of XMRV RNA in plasma had declined to <1 copy/mL in both animals. PBMC-associated XMRV DNA was also measured by X-SCA. DNA levels peaked with similar kinetics as plasma viremia but persisted with levels of 23 and 645 copies/10^6^ PBMC in the two animals, respectively, at the end of the follow-up period, 119 days after inoculation. Antibody reactivity to XMRV capsid (CA) and transmembrane protein (TM) measured by ELISA was undetectable prior to inoculation but were robustly positive thereafter (Table 2) (Del Prete et al., in preparation). Replication competent XMRV cannot be cultured from macaque plasma or PBMC samples due to extensive hypermutation of the provirus post-inoculation, likely due to the effect of APOBEC proteins (Del Prete et al., in preparation). Consequently, XMRV-spiked human plasma was used to verify the DERSE.L-iG-P cells for detection of XMRV.

### 3.4. Testing Prostate Cancer Samples for XMRV Nucleic Acid, Antibodies, and Isolatable Virus

**Figure 3:**
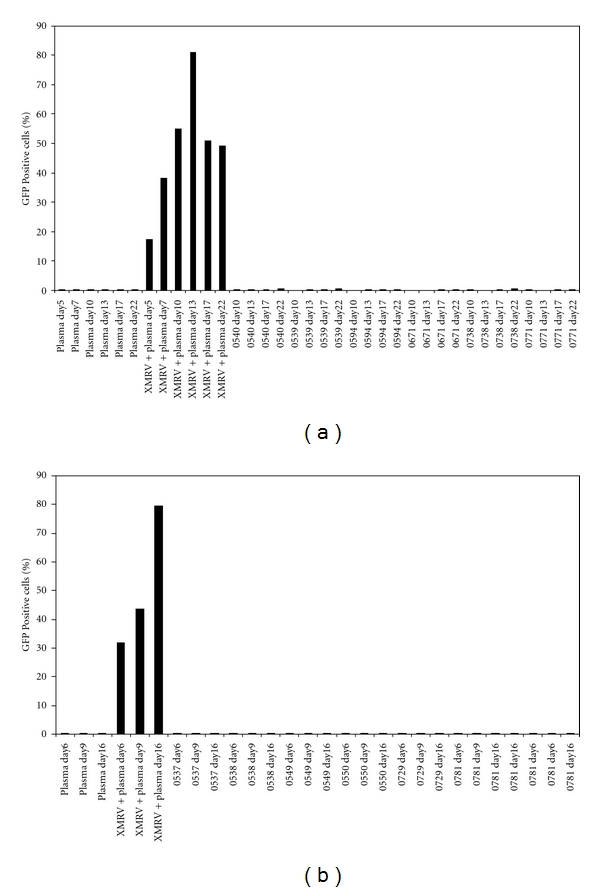
*Testing plasma samples from prostate cancer patients for replication competent XMRV.* Twelve samples were blinded as to their X-SCA and ELISA results and were tested for replicating virus using the DERSE.L-iG-P assay in two separate experiments. Six samples were tested in experiment 1 at passages 10, 13, 17, and 22 (a). All passages were negative for XMRV while virus was recovered from the positive control samples (10^7^ copies of XMRV from 22Rv1 cells spiked into human plasma). Six additional samples were tested in experiment 2 at passages 6, 9, and 16 (b). All passages were negative for XMRV while virus was recovered from the positive control samples.

Samples obtained from the two cohorts of prostate cancer patients were assayed first for XMRV nucleic acid (X-SCA) and antibody reactivity against XMRV CA and TM protein (Tables 3 and 4). No plasma or prostate tissue samples in the NIH prostate cancer cohort or the UC Davis prostate cancer cohort were positive for XMRV nucleic acids or antibodies (Tables 3 and 4). However, two plasma samples in the NIH cohort (0594, 0771) were indeterminate for XMRV RNA. One of these samples (0594) was negative by ELISA, and the other (0771) had an indeterminate ELISA result. One other patient sample in the NIH cohort (0781) was indeterminate for XMRV antibody reactivity but negative for XMRV nucleic acid (Table 3). All three of these samples, along with 9 matched negative samples, were blinded and tested for replicating virus using the DERSE.L-iG-P assay. Virus could not be cultured from any of these plasma samples while it was readily recovered from positive control samples (22Rv1-derived XMRV spiked into negative human plasma) (Figure 3). Consequently, by our prospectively defined criteria, none of the 26 patient samples in the NIH cohort were considered to be XMRV infected (positive for nucleic acid, antibody, and/or replication competent virus) (Table 3). All 108 plasma samples from prostate cancer patients obtained from UC Davis were assayed for XMRV RNA and antibodies (Table 4). All samples were negative for XMRV nucleic acid except one (0739), which was indeterminate. No sample was found to be antibody reactive by our ELISA criteria (at least 50% reactive relative to the macaque positive control sera). Twelve of the 108 samples were indeterminate for XMRV reactivity to either CA or TM (2 standard deviations above the average negative human sample) but were negative for nucleic acid (Table 4). No sample was indeterminate or positive for both XMRV nucleic acid and antibody, and therefore, all were determined to be negative for XMRV infection.

## 4. Discussion

After publication of the XMRV study by Lombardi et al. in October 2009 suggesting a possible disease association with CFS and a surprisingly high apparent seroprevalence for XMRV even among healthy control subjects, researchers at the NCI-Frederick set out to develop rigorous methods to evaluate the prevalence of XMRV infection. Using control samples, including spiked specimens where appropriate, we developed assays to measure plasma XMRV RNA viremia, cell-associated XMRV DNA levels, and antibodies to XMRV CA and TM. Because Lombardi et al. reported the presence of culture rescuable replication-competent virus from the blood of study subjects using coculture with a human cell line (LNCap), we created DERSE cells, derivatives of the same LNCap cells with a fluorescent reporter to detect XMRV replication. These cells broadly and sensitively detect the replication of different MLV-related gammaretroviruses that exhibit a tropism for human prostate cancer cells. In the absence of patient-derived definitive positive and negative control specimens, we applied our different assay methods to samples obtained from two pigtail macaques prior to and after experimental XMRV inoculation. XMRV plasma viremia was detectable in both inoculated macaques for 2-3 weeks after inoculation but then declined to undetectable levels (Del Prete et al., in preparation). However, XMRV DNA in PBMCs and serum antibodies remained at readily measurable levels for the duration of study follow-up in both animals (Del Prete et al., in preparation). Evaluation of samples from the inoculated macaques demonstrated the ability of our methods to reliably detect evidence of XMRV infection in blood samples and showed that XMRV provirus and antibodies persist even when viremia is not detectable.

 In the development of diagnostic tools for XMRV infection, it became clear that a single method for XMRV detection would not be sufficient for definitive diagnosis due to a high frequency of false positives by PCR from contaminating nucleic acids (especially mouse genomic DNA) and high background reactivity seen by ELISA, even in samples from healthy control subjects, presumably reflecting cross-reactivity. Therefore, we suggest a multiple assay approach to determine the XMRV status of patient samples. We established diagnostic criteria requiring that all replicates from X-SCA analysis must be positive and that serum antibodies and/or replicating virus must also be detectable in the same patient in order to report the patient XMRV positive. Samples resulting in discordant results from PCR replicates are reported as indeterminate. Despite earlier reports that evidence of XMRV infection was detected in as many as 20% of prostate tumors [2, 10–12], using the assays we developed, we did not find clear evidence for XMRV in the blood of two independent cohorts of patients with prostate cancer (total *n* = 134) or in the prostate tissue of a small subset of these individuals (*n* = 19). Based on previously reported frequencies of XMRV detection in prostate cancer patients, if XMRV is present in the blood of infected individuals, we expected that approximately 27 of the 134 patients in our study would be positive for XMRV. One patient from the NIH cohort (0771) had an indeterminate X-SCA result (2/3 reactions were positive for RNA). This sample was also positive for reactivity to CA and TM by ELISA. However, no XMRV DNA was found in the whole blood from this patient, and replication competent virus could not be recovered from the sample. Taken together, these data are considered an indeterminate result by our criteria. No other samples were positive by more than one diagnostic method.

 The occasional positive X-SCA reaction is not above background for this assay. We regularly run 96-well plates of “no template controls” using both our X-SCA primers and primers targeting intracisternal A particles (IAP) [20, 21, 33] that are present in high copies in the mouse genome in order to monitor the levels of contaminating mouse DNA in the reagents and in the environment. We have found that about 5% of wells are positive with the X-SCA primers and about 20% with the IAP primers. Based on these backgrounds, we expect to detect low levels of mouse DNA contamination in samples tested, as seen is this study and in others [20, 21, 33]. Therefore, we required that all replicates of patient samples be positive to obtain a “positive” X-SCA result. We did not test the samples directly with IAP primers since we have not successfully found reagents and an environment that are free from mouse genomic DNA (on average about 1/3000 of a mouse genome per PCR reaction).

 Although we had an occasional indeterminate result for XMRV RNA in the plasma samples studied, we did not detect XMRV DNA in any sample tested, despite the ability of our assay to sensitively detect XMRV DNA in spiked control samples and in specimens from inoculated macaques [28] (Del Prete et al., in preparation). Results from the inoculated macaques showed that in experimental infection, XMRV proviral DNA is readily measurable in blood cells even when plasma viremia was not detectable (Del Prete et al., in preparation), further suggesting that these patients do not carry XMRV in their blood. Findings from previous studies reporting higher prevalence for XMRV in similar cohorts [2, 11, 12] typically involved testing of prostate tumors. None of these studies reported the detection of XMRV in blood samples or the isolation of infectious virus from clinical specimens, and only one measured the presence of reactive antibodies through a virus neutralization assay [10]. Detection of antibody responses to specific viral proteins by ELISA or by reactivity to XMRV immunoblots was not assessed. If we had used less rigorous criteria basing an overall diagnosis on a single, nonconfirmed test and not requiring all replicates to yield the same result, then our two cohorts would have given rise to an apparent, and in our view almost certainly incorrect, reported XMRV prevalence rate of approximately 12%. These considerations may explain conflicting prior reports for the prevalence of XMRV and are consistent with claims that XMRV detection is likely the result of laboratory contamination [22, 26, 33, 34]. Particularly given the potential for false positive results in PCR and serological assays for XMRV, our results suggest that applying multiple diagnostic methods including measuring levels of proviral DNA in blood cells provides a more reliable approach for investigating the prevalence of XMRV. These results also demonstrate that XMRV nucleic acid, and antibodies are undetectable in the blood of patients with prostate cancer.

## Figures and Tables

**Table 1:** X-SCA Results on XMRV-inoculated macaques.

Monkey ID	Days after inoculation	Plasma XMRV RNA copies/mL	Copies of XMRV DNA per 10^6^ PBMCs
14232	0	<1.1	0
14232	5	534.11	197
14232	119	<1.1	23

8242	0	<1.1	0
8242	13	2153.56	2833
8242	119	<1.1	645

**Table 2:** Immunoassay of plasma from XMRV-inoculated macaques.

Monkey ID	Days after inoculation	Reactivity with	
		CA	TM
8242	0	19.5	248.5
8242	76	12713	544405

14232	0	14.5	145
14232	76	20108	285277

**Table 3:** X-SCA, ELISA, and virus culture results on prostate cancer samples from NIH cohort.

			Nucieic acid testing by X-SCA				Serologic Testing by ELISA			Virus Culture	
Sample number	Sample ID	Date of blood draw	Plasma XMRV RNA copies/mL	XMRV DNA copies/mL in whole blood	XMRV DNA copies in prostate tissue	Number of prostate cells tested	CA	TM	ELISA result	Virus replication	Overall result
1	UB10-0533	8/5/2010	<0.6	<10	0	174000	999	985	NR	NT	NEGATIVE
2	UB10-0537	8/6/2010	<0.8	<10	NA	NA	5713	5302	NR	NEGATIVE	NEGATIVE
3	UB10-0538	8/6/2010	<0.7	<10	NA	NA	2323	2362	NR	NEGATIVE	NEGATIVE
4	UB10-0539	8/6/2010	<0.8	<0.9	NA	NA	1505	1864	NR	NEGATIVE	NEGATIVE
5	UBI0-0540	8/6/2010	<0.8	<0.9	3.8	105600	1429	2811	NR	NEGATIVE	NEGATIVE
6	UB10-0542	8/9/2010	<0.7	<10	0	307500	1796	1949	NR	NT	NEGATIVE
7	UBI0-0547	8/11/2010	<0.8	<10	0	81000	2248	4325	NR	NT	NEGATIVE
8	UBI0-0548	8/11/2010	<0.8	<10	NA	NA	2566	2826	NR	NT	NEGATIVE
9	UBI0-0549	8/11/2010	<0.8	<10	0	59025	5129	6059	NR	NEGATIVE	NEGATIVE
10	UB10-0550	8/11/2010	<0.8	<10	0	54825	1412	1414	NR	NEGATIVE	NEGATIVE
11	UB10-0578	8/19/2010	<0.8	<10	0	275250	1,412	1,460	NR	NT	NEGATIVE
12	UB10-0594	8/21/2010	*0.9*	<0.9	0	144075	3,050	3,190	NR	NEGATIVE	NEGATIVE
13	UB10-0643	9/14/2010	<0.7	<10	0	32400	5.359	5.430	NR	NT	NEGATIVE
14	UB10-0665	9/16/2010	<0.7	<10	0	84300	2.736	3.817	NR	NT	NEGATIVE
15	UBl0-0671	9/21/2010	Invalid test	<0.9	NA	NA	1,490	1,375	NR	NEGATIVE	NEGATIVE
16	UB10-0706	9/28/2010	Invalid test	<10.0	0	3502.5	2.514	2.500	NR	NT	NEGATIVE
17	UB10-0729	9/30/2010	<0.7	<10.0	0	88950	1,453	1,620	NR	NEGATIVE	NEGATIVE
18	UB10-0771	10/14/2010	*10.4*	<0.9	NA	NA	10.655	26.030	*Equiv*	NEGATIVE	Indeterminate
19	UBl0-0781	10/15/2010	<0.7	<10	NA	NA	8.151	8.451	*Equiv*	NEGATIVE	NEGATIVE
20	UB10-0738	10/5/2010	Invalid test	<0.9	0	74325	2.280	2.312	NR	NEGATIVE	NEGATIVE
21	UB10-0785	10/18/2010	<16.5	<10	0	27375	3.840	3.244	NR	NT	NEGATIVE
22	UB10-0788	10/19/2010	<16.5	<10	0	20887	2.990	2.596	NR	NT	NEGATIVE
23	UBl0-0830	10/29/2010	<0.8	<10	0	63225	NT	NT	NT	NT	NEGATIVE
24	UB10-0833	11/1/2010	<0.8	<10	0	41175	NT	NT	NT	NT	NEGATIVE
25	UBl0-0853	11/4/2010	<0.8	<10	0	8535	NT	NT	NT	NT	NEGATIVE
26	UB10-0913	11/19/2010	<0.8	<10	0	7132	NT	NT	NT	NT	NEGATIVE

NA: sample not available.

NT: sample not tested.

**Table 4:** X-SCA and ELISA results on prostate cancer samples from UC-Davis cohort.

Plasma RNA				Plasma RNA			
Patient ID	Copies/mL	ELISA result	Overall result	Patient ID	Copies/mL	ELISA result	Overall result
P0005	<16.5	*Indeterminate*	NEGATIVE	P0566	<16.5	NR	NEGATIVE
P0013	<16.5	NR	NEGATIVE	P0572	<16.5	NR	NEGATIVE
P0015	<16.5	NR	NEGATIVE	P0592	<16.5	NR	NEGATIVE
P0024	<16.5	NR	NEGATIVE	P0593	<16.5	NR	NEGATIVE
P0026	<16.5	NR	NEGATIVE	P0605	<16.5	NR	NEGATIVE
P0027	<16.5	NR	NEGATIVE	P0611	<16.5	NR	NEGATIVE
P0031	<16.5	NR	NEGATIVE	P0612	<16.5	NR	NEGATIVE
P0034	<16.5	NR	NEGATIVE	P0617	<16.5	NR	NEGATIVE
P0036	<16.5	NR	NEGATIVE	P0632	<16.5	NR	NEGATIVE
P0044	<16.5	NR	NEGATIVE	P0637	<16.5	NR	NEGATIVE
P0045	<16.5	NR	NEGATIVE	P0641	<16.5	NR	NEGATIVE
P0118	<16.5	NR	NEGATIVE	P0650	<16.5	NR	NEGATIVE
P0133	<16.5	NR	NEGATIVE	P0657	<16.5	*Indeterminate*	NEGATIVE
P0144	<16.5	NR	NEGATIVE	P0659	<16.5	NR	NEGATIVE
P0154	<16.5	NR	NEGATIVE	P0672	<16.5	NR	NEGATIVE
P0156	<16.5	NR	NEGATIVE	P0673	<16.5	NR	NEGATIVE
P0162	<16.5	*Indeterminate*	NEGATIVE	P0675	<16.5	NR	NEGATIVE
P0167	<16.5	NR	NEGATIVE	P0679	<16.5	NR	NEGATIVE
P0170	<16.5	NR	NEGATIVE	P0685	<16.5	NR	NEGATIVE
P0172	<16.5	NR	NEGATIVE	P0710	<16.5	NR	NEGATIVE
P0177	<16.5	NR	NEGATIVE	P0721	<16.5	NR	NEGATIVE
P0185	<16.5	NR	NEGATIVE	P0723	<20.6	NR	NEGATIVE
P0195	<16.5	*Indeterminate*	NEGATIVE	P0726	<16.5	*Indeterminate*	NEGATIVE
P0209	<16.5	NR	NEGATIVE	P0733	<16.5	NR	NEGATIVE
P0219	<16.5	*Indeterminate*	NEGATIVE	P0739	*55*	NR	NEGATIVE
P0232	<16.5	NR	NEGATIVE	P0766	<20.6	NR	NEGATIVE
P0239	<16.5	NR	NEGATIVE	P0778	<16.5	NR	NEGATIVE
P0293	<16.5	NR	NEGATIVE	P0787	<16.5	NR	NEGATIVE
P0306	<16.5	NR	NEGATIVE	P0792	<16.5	NR	NEGATIVE
P0314	<16.5	NR	NEGATIVE	P0826	<16.5	NR	NEGATIVE
P0321	<16.5	NR	NEGATIVE	P0846	<16.5	NR	NEGATIVE
P0322	<16.5	NR	NEGATIVE	P0848	<16.5	NR	NEGATIVE
P0325	<16.5	NR	NEGATIVE	P0852	<20.6	NR	NEGATIVE
P0327	<16.5	NR	NEGATIVE	P0906	<16.5	*Indeterminate*	NEGATIVE
P0332	<16.5	*Indeterminate*	NEGATIVE	P0916	<16.5	NR	NEGATIVE
P0340	<16.5	*Indeterminate*	NEGATIVE	P0923	<16.5	NR	NEGATIVE
P0342	<16.5	NR	NEGATIVE	P0952	<16.5	NR	NEGATIVE
P0346	<16.5	*Indeterminate*	NEGATIVE	P0984	<16.5	NR	NEGATIVE
P0348	<16.5	NR	NEGATIVE	P0989	<16.5	NR	NEGATIVE
P0351	<16.5	NR	NEGATIVE	P0996	<16.5	NR	NEGATIVE
P0355	<16.5	NR	NEGATIVE	P0999	<16.5	NR	NEGATIVE
P0366	<20.6	NR	NEGATIVE	P1010	<16.5	NR	NEGATIVE
P0380	<16.5	NR	NEGATIVE	P1025	<16.5	NR	NEGATIVE
P0382	<16.5	NR	NEGATIVE	P1032	<16.5	NR	NEGATIVE
P0384	<16.5	NR	NEGATIVE	P1063	<16.5	NR	NEGATIVE
P0388	<16.5	NR	NEGATIVE	P1076	<16.5	NR	NEGATIVE
P0509	<16.5	*Indeterminate*	NEGATIVE	P1086	<16.5	NR	NEGATIVE
P0511	<16.5	NR	NEGATIVE	P1108	<16.5	*Indeterminate*	NEGATIVE
P0530	<16.5	NR	NEGATIVE	P1110	<16.5	NR	NEGATIVE
P0532	<16.5	NR	NEGATIVE	P1211	<16.5	NR	NEGATIVE
P0535	<16.5	NR	NEGATIVE	P1268	<16.5	NR	NEGATIVE
P0536	<16.5	NR	NEGATIVE	P1297	<16.5	NR	NEGATIVE
P0544	<16.5	NR	NEGATIVE	P1304	<16.5	NR	NEGATIVE
P0562	<16.5	NR	NEGATIVE	P1318	<16.5	NR	NEGATIVE
